# The influence of environmental conditions on safety management in hospitals: a qualitative study

**DOI:** 10.1186/s12913-018-3116-8

**Published:** 2018-05-02

**Authors:** Carien W. Alingh, Jeroen D. H. van Wijngaarden, Robbert Huijsman, Jaap Paauwe

**Affiliations:** 1 Erasmus School of Health Policy & Management, Erasmus University Rotterdam, P.O. Box 1738, 3000 DR Rotterdam, The Netherlands; 2 Department of Human Resource Studies, Tilburg University, P.O. Box 90153, 5000 LE Tilburg, The Netherlands

**Keywords:** Patient safety, Hospital management, Control, Commitment

## Abstract

**Background:**

Hospitals are confronted with increasing safety demands from a diverse set of stakeholders, including governmental organisations, professional associations, health insurance companies, patient associations and the media. However, little is known about the effects of these institutional and competitive pressures on hospital safety management. Previous research has shown that organisations generally shape their safety management approach along the lines of control- or commitment-based management. Using a heuristic framework, based on the contextually-based human resource theory, we analysed how environmental pressures affect the safety management approach used by hospitals.

**Methods:**

A qualitative study was conducted into hospital care in the Netherlands. Five hospitals were selected for participation, based on organisational characteristics as well as variation in their reputation for patient safety. We interviewed hospital managers and staff with a central role in safety management. A total of 43 semi-structured interviews were conducted with 48 respondents. The heuristic framework was used as an initial model for analysing the data, though new codes emerged from the data as well.

**Results:**

In order to ensure safe care delivery, institutional and competitive stakeholders often impose detailed safety requirements, strong forces for compliance and growing demands for accountability. As a consequence, hospitals experience a decrease in the room to manoeuvre. Hence, organisations increasingly choose a control-based management approach to make sure that safety demands are met. In contrast, in case of more abstract safety demands and an organisational culture which favours patient safety, hospitals generally experience more leeway. This often results in a stronger focus on commitment-based management.

**Conclusions:**

Institutional and competitive conditions as well as strategic choices that hospitals make have resulted in various combinations of control- and commitment-based safety management. A balanced approach is required. A strong focus on control-based management generates extrinsic motivation in employees but may, at the same time, undermine or even diminish intrinsic motivation to work on patient safety. Emphasising commitment-based management may, in contrast, strengthen intrinsic motivation but increases the risk of priorities being set elsewhere. Currently, external pressures frequently lead to the adoption of control-based management. A balanced approach requires a shift towards more trust-based safety demands.

## Background

Healthcare organisations are confronted with increasing safety demands from a diverse set of stakeholders [1], including governmental organisations, professional associations, health insurance companies, patient associations and the media. In this multidimensional or layered environment hospitals have to deal with various coexisting institutional and competitive pressures [2, 3]. The systems approach claims that these environmental conditions influence the shaping of organisational policies and procedures, which affect the work processes of healthcare professionals who try to provide the safest possible care to their patients [4]. However, little empirical research has been done on the actual consequences of various environmental conditions for safety management in healthcare [2].

Previous research has shown that organisations generally shape their safety management approach along the lines of control- or commitment-based management [5, 6]. The former is a formalised, top-down approach that focuses on regulating work processes, monitoring professional behaviours and providing employees with feedback on their level of compliance [7, 8]. In contrast, commitment-based management focuses on facilitating an internalization of safety norms and values in employees [6, 9], by creating awareness of safety risks, stressing the priority of safety within the organisation and encouraging employees’ ownership in safety management [5]. Each approach might have its merits in optimising safety [10], and both may be required in professional organisations such as hospitals.

To understand the relationship between environmental conditions and organisations’ management approach, Paauwe developed the contextually-based human resource (HR) theory [11, 12]. This framework describes how environmental conditions influence the shaping of HR management, incorporating institutional pressures, competitive drivers, and the historically grown configuration of an organisation. Moreover, it combines a systems approach with an actor perspective that stresses the role of strategic agency within organisations. Depending on the room to manoeuvre that organisations experience, the individuals or groups who hold decision-making power within the organisation (i.e.*,* the dominant coalition) may opt for various strategically chosen responses while shaping management policies and procedures [13]. In this article we will adapt this framework to patient safety, since environmental conditions and strategic responses of organisations are considered to be issue-specific [14].

Management policies and practices are, first, subject to the influences of institutional mechanisms. Institutions reflect sets of rules, norms or belief systems which provide stability and meaning to social life [15], and which are “*the rules of the game”* that direct and control organisational behaviour [16]. According to new institutionalism [17], organisations conform to these institutional pressures in order to gain legitimacy and to improve their chances of survival [18, 19]. As a consequence organisations acting in similar contexts become more and more homogeneous. This isomorphic change results from three mechanisms [17]. First, *coercive* mechanisms derive from cultural expectations in society and (in)formal pressures from institutions on which the organisations are dependent. Prototypically, stakeholders such as governmental agencies demand organisations to adopt specific practices and have the ability to punish non-compliance. Second, *mimetic* mechanisms originate from uncertainty which drives organisations towards imitating practices of successful competitors or ‘best practices’. Finally, *normative* mechanisms arise from professionalization as professional networks and training programs develop and spread professional norms and values.

Whereas seeking legitimacy may drive organisations towards institutional isomorphism, an economic rationality of efficiency and effectiveness, may steer organisations either in the direction of competitive isomorphism or towards differentiation. Exposure to similar market conditions and endeavours to improve efficiency or to keep up with competitors may lead to similarities in organisational practices and systems [17]. Organisations may, for example, benchmark themselves against each other and imitate competitors’ policies and practices which are promising for delivering desirable outcomes. However, strategic management scholars advocate that organisations should ‘be different’ in order to gain a competitive advantage [20, 21]. The transition to regulated competition through market-oriented healthcare reforms forces hospitals to compete on both quality and price, which may stimulate them to differentiate based on safety management and performance.

In addition to influences of institutional and competitive mechanisms, the historically grown configuration of an organisation has a role in shaping management policies and practices as well [11]. The configuration reflects a unique path-dependent pattern of organisational characteristics, structures, competences and values, which is also referred to as the administrative heritage [22]. According to Delery & Doty’s configurational approach, organisations need to align their management policies and practices with the administrative heritage in order to be effective [23]. Veld studied the historical configuration of hospitals in the Netherlands and found that it is characterised by ongoing mergers and reorganisations, a highly professionalised workforce, status differences between disciplines, and the autonomous position of medical specialists [24]. In the Netherlands, the majority of medical specialists are, for example, employed in independent partnerships and hold a relatively independent position in the managerial hierarchy, making it hard to control their behaviours. Nevertheless, they have considerable formal and informal power in hospital policy and management, since the hospital needs their commitment in order to achieve its objectives.

How the dominant coalition deals with these environmental conditions depends on the room to manoeuvre or leeway that organisations experience to opt for various strategic responses. The dominant coalition may mitigate the relationship between environmental conditions and the organisation by obtaining a degree of leeway for shaping management policies and practices. This room to manoeuvre is affected by several factors, including the financial health of the organisation [25], the dependency relationships with external stakeholders [13], and actors’ sense-making of environmental pressures and their interpretation of what is considered appropriate behaviour [26]. Moreover, internal dynamics in the dominant coalition in terms of interests, values and power dependencies may also influence the room to manoeuvre to make strategic choices [27]. According to the strategic balance theory [28], organisations make strategic choices *“to be [either] more differentiated from or more similar to its competitors”* in order to achieve a balance between requirements of stakeholders, pressures for legitimization and competition [29]. Hence, although institutional pressures have the power to force organisations to adopt certain practices, actors within the organisation still have ample room to enact agency [30]. Oliver distinguishes five manifestations of organisational agency [13]. First, organisations could passively conform to institutional requirements. Second, under conditions of conflicting demands or inconsistencies between external expectations and internal objectives, organisations could compromise by balancing or bargaining the demands. Moreover, they may choose to buffer or decouple themselves from institutional pressure by ‘ceremonial’ implementation; pretending conformity without true believe or shared values by the members of the organisation [19]. In other words, ceremonial implementation concerns relatively high levels of implementation accompanied by low levels of internalisation [14]. The fourth strategic response is a more active form of resistance in which organisations ignore, challenge or attack institutional norms and expectations. And finally, organisations may choose to manipulate demands by a purposeful and opportunistic attempt to co-opt, influence, or control institutional pressures [13]. Formulated in a more positive way, they have the opportunity to ‘lead’, ‘initiate’ or ‘develop’ strategic responses to environmental demands [11], or they may seek to bring about institutional change; also referred to as institutional entrepreneurship [31]. Hence, actors within an organisation who have an interest in particular institutional arrangements may exercise power and attempt to actively transform existing institutional arrangements and create new ones.

**Fig. 1:**
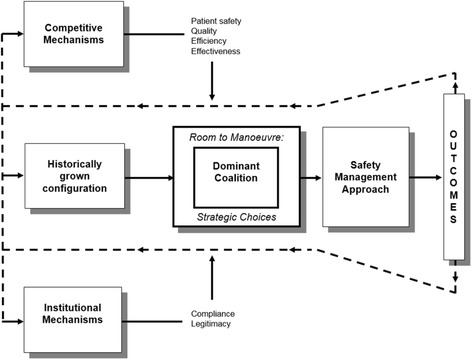
Heuristic framework, based on the contextually-based HR theory [11, 12]

The aforementioned organisational responses imply that, in the end, the dominant coalition makes strategic decisions; thus, shaping management policies and practices. The current study aims to develop a deeper understanding how the combination of institutional, competitive and configurational factors as well as internal issues of strategic choice influences the shaping of safety management approaches of healthcare organisations. During a qualitative study conducted in five hospitals in the Netherlands, Paauwe’s contextually-based HR theory is used as a heuristic framework (see Fig. 1) [11, 12].

## Methods

We selected five hospitals in the Netherlands, based on organisational characteristics as well as their variation in reputation for patient safety. We interviewed hospital managers and staff with a central role in safety management. According to the Dutch law, no ethical approval was required from a Medical Ethical Committee [32].

### Research setting

Hospital care in the Netherlands is delivered in private, not-for-profit care organisations. Since the introduction of the Health Insurance Act in 2006, the organisations are subject to a system of so-called regulated competition. On the one hand, health insurers purchase healthcare and negotiate with providers on both quality and price, while on the other hand the government governs at a distance in order to guarantee universal access to high-quality care [33]. As a result, hospitals are subject to a wide variety of requirements which may influence how they manage patient safety.

In 2013, a total of 89 Dutch hospitals existed, which could be categorized into university medical centres, top-clinical teaching hospitals and general hospitals [34]. A combination of general and top-clinical teaching hospitals were considered for inclusion in the study (see Table 1); university medical centres were excluded because of the great degree of organisational complexity of these organisations (including research and education). Moreover, variation was sought in hospital size as well as organisations’ safety performances. Performance scores were derived from publicly available ranking lists (i.e., Elsevier rankings) and consisted of a combined score of various safety performance indicators (e.g., process indicators on patient identification and the screening of pressure ulcers). Since the ranking lists have been criticised for fluctuation over time [35], the scores of three successive years have been combined. The five participating hospitals were selected using stratified purposeful sampling [36], and provided a reflection of the variation in hospital size and safety reputation across all Dutch general and top-clinical teaching hospitals.

**Table 1:** Case characteristics of the five hospitals

	Hospital A	Hospital B	Hospital C	Hospital D	Hospital E
Type of hospital	Top-clinical	Top-clinical	General	General	Top-clinical
Hospital size (no. of beds)	< 500	750–1000	500–750	500–750	> 1000
Safety performance*	Low	Low	Low	Mediocre	High

*Safety performance has been reported on a scale that ranges from 1 to 4. Scores < 2 are indicated as low, scores of 2–3 are indicated as mediocre and scores > 3 are indicated as high

### Data collection

In order to gain deep insights into the phenomenon of interest, semi-structured interviews were conducted with respondents who occupy a central role in safety management and who work at different hierarchical levels within the organisation [37]. From September 2013 to April 2014, a total of 43 interviews were conducted with 48 respondents (some interviews were duo-interviews), including (chief) patient safety officers, members of the board of directors, members of the medical advisory board, medical managers, business unit managers and nurse managers or team leaders (see Table 2). All of the respondents were (directly) involved in safety management and could give insight into the reasons underlying the choice for different safety management approaches. By purposefully selecting respondents who hold different managerial positions and work at different hierarchical levels we aimed to gain broad insight into varying viewpoints in the dominant coalition on how internal and external contextual features combine to influence the shaping of safety management approaches across hierarchical levels. After all, how strategic-level managers respond to institutional, competitive and configurational factors might differ from the choices made by managers at tactical or operational hospital levels.

**Table 2:** Number of respondents per function

	Hospital A	Hospital B	Hospital C	Hospital D	Hospital E	Total
1. (Chief) patient safety officer	1	2	3	1	1	8
2. Board of directors	1	1	1	1	1	5
3. Medical manager/advisory board	2	2	2	4	4	14
4. Business unit manager	2	2	1	0	2	7
5. Nurse manager/team leader	4	2	2	3	3	14
Total	10	9	9	9	11	48

The interviews were structured around the constructs underlying the contextually-based HR theory [11, 12]. Respondents were, first, asked to describe how patient safety is managed and what safety interventions are applied in their department or hospital. Subsequently, the interview addressed environmental conditions and relevant trends in the hospital context that might have influenced the safety management approach. Respondents were, for example, asked *what* developments took place in the healthcare context (e.g., institutional or competitive mechanisms) or in their own organisation that might have influenced how they manage patient safety. In addition, the interview focused on *how* these developments affected the safety management approach and how organisations respond to environmental conditions; in other words, did hospitals experience room to manoeuvre? Finally, respondents were asked to elaborate on *why* hospitals opted for specific strategic responses in reaction to demands from stakeholders in their environment.

### Data analysis

All interviews were audio-recorded and transcribed verbatim. The transcripts were analysed using qualitative data analysis software Atlas.ti to conduct a thematic analysis. First, the researchers familiarised themselves with the data by (re)reading transcripts and identifying *“patterns of meaning and issues of potential interest in the data”* [38]. Second, initial codes were generated to identify topics of interest. To identify codes, deductive- and inductive-coding were combined. The initial list of codes consisted of key-elements of the conceptual framework [11, 12], and included codes such as ‘competitive mechanisms’, ‘dominant coalition’, and ‘room to manoeuvre’. However, the researchers remained open for codes that emerged from the data and searched for specifications of initial codes. For example, the initial code ‘competitive mechanisms’ covered elements such as ‘purchasing healthcare by insurance companies’, ‘publically available ranking lists’ and ‘benchmarking’. Whereas the initial code ‘room to manoeuvre’ was further specified by factors which influence the experienced leeway, such as ‘tightness of external supervision’ and ‘relevance of safety requirements’. Furthermore, new codes emerged from the data, such as ‘critical safety incidents’. In the end, all codes were combined into broader (sub)themes, which were based on similarities in data as well as theory. The final themes structure the results presented in this paper. In the results section, respondents are referred to as codes corresponding with the letters and numbers for hospitals and functions as mentioned in Table 2.

## Results

### Dominant coalition shapes safety management

Although the formal responsibility rests with the board of directors, all hospitals in this study established a structure of shared responsibilities and joint decision-making on hospital-wide safety policies and practices: *“Together with the board of directors, the medical advisory board takes decisions on many organisational issues. For all topics related to the national programme ‘Prevent Harm, Work Safely’, an action plan is, for example, presented which is approved by both of them”* (C1). Medical specialists have a powerful voice in these decision-making processes, especially in case of care-related matters such as patient safety. *“There is no board of directors of a Dutch hospital who does something that doctors don’t want to, because then your days as a board member are simply numbered”* (A3). Remarkably, nurses, who have a central role in care delivery and who form a significant part of the hospital staff, are not closely involved in shaping hospital-wide safety policies and practices.

With regard to departmental safety issues, a similar pattern of shared responsibilities was found. *“Together with the medical manager, as a duo we are responsible for taking care of and ensuring patient safety [in our department]”* (E4). Departmental safety policies and practices are deeply influenced by choices made at the hospital level. Nonetheless, business unit managers, medical managers and nurse managers still have some leeway for shaping safety management within their own department.

### Institutional demands

The studied hospitals are subject to coercive pressures resulting from requirements and expectations of the Dutch Healthcare Inspectorate, safety legislations, government initiatives and accreditation committees. The Dutch Healthcare Inspectorate has, for example, the authority to keep hospitals under ‘stringent supervision’ or even close a department or organisation that does not meet safety requirements. *“If the inspectorate takes steps to enforce compliance and you do not follow a guideline […], they say you do not work safely or you work on the brink of what is considered acceptable. Then the inspectorate enforces you to improve things within a month, or the department will be closed”* (B1). In line with this, the inspectorate supervises hospitals by undertaking site visits and by discussing safety performance indicators which provide insight into the safety of care processes.

Rather than punishing non-compliance, hospitals may also be forced in more subtle ways to meet safety requirements. For example, hospital accreditations let independent committees check whether hospitals comply with a set of (minimum) safety standards. These accreditations shifted from voluntary participation to a required standard in order to gain legitimacy in the hospital field. Something similar is the case for the national programme ‘Prevent Harm, Work Safely’ which was a joint initiative of the government and professional associations, offering hospitals tools and best practices for certain high-risk patient safety problems like surgical site infections or medication errors. Whereas the programme was primarily intended to encourage safety improvement, hospitals were eventually expected to adopt specific practices and to reach accreditation on how they manage safety risks. *“When you combine the national programme ‘Prevent Harm, Work Safely’ with a system of auditing and accrediting hospitals, there is no escape anymore”* (D2). So, the choices of the dominant coalition are, first of all, influenced by coercive pressures resulting from expectations of the organisational field and demands from stakeholders that have the ability to enforce certain safety behaviours.

Secondly, safety management is also influenced by normative mechanisms deriving from professional norms and regulations. In professional training programmes, healthcare professionals are socialised to strive for safe care, to work fairly independent of external control mechanisms and to rely on self-judgement. As a result, *“Every doctor is convinced that he delivers high-quality care and that he works safely. […] It is a very isolated world, the medical world”* (B3). Moreover, medical professional associations establish evidence-based clinical protocols and guidelines on how to deliver safe care: *“All rules of the game concerning patient safety are established by our professional associations, […] for example on how to apply hand hygiene”* (B1). These normative regulations do not only contribute to safety management in itself, some of them are also adopted by the Healthcare Inspectorate or accreditation committees which enforce compliance with the protocols or guidelines.

Finally, the studied hospitals do also use mimetic mechanisms by seeking inspiration from other high-risk industries while shaping safety management. For example, different hospitals are inspired by successful initiatives from aviation or petrochemical industry. *“One of the actions that is currently taken is that I will try to find a way to change the speaking up culture together with the guy who is running the speaking up project at Shell”* (E3).

### Competitive mechanisms

The choices made by the dominant coalition are also affected by competitive mechanisms deriving from the healthcare market. First, health insurers play a major role in the healthcare market, since they negotiate with hospitals on both quality and price of the care that is provided: *“They [health insurers] do not purchase certain types of care if you do not meet their quality standards”* (C2). As a result of the dominance of health insurers, hospitals typically experience little leeway to deviate from their safety requirements. Even though, hospitals generally experience that insurers mostly focus on financial aspects and cost reduction: *“Health insurers state that quality and safety are really important, but in the meantime they negotiate till there is no meat left on the bone”* (C1). As a consequence, hospitals are on the one hand stimulated to focus on patient safety, while on the other hand they experience limited financial resources to allocate to safety management.

In addition, hospitals do also feel a sense of urgency to work on patient safety because patients become better informed and critical customers, since news and social media report on serious safety incidents, patient experiences and ranking lists on hospitals’ quality and safety. A bad reputation of a hospital reflects badly on the professionals involved: *“Doctors don’t like to explain at a birthday party why they, as a hospital, are number 88 [in a top 100 ranking list]”* (B3). Negative publicity may also have more serious consequences in the current Dutch market system: *“If we do not provide good care we will not get any clients or patients. Then the hospital will earn no money”* (C3).

Thirdly, safety management is also influenced by inspiration drawn from comparisons with competitors. Although benchmarking patient safety data is not yet common sense on hospital level, some intensive care units and surgical departments do compare their safety processes and outcomes with similar departments in other hospitals, sometimes even internationally. *“Especially in orthopaedics, infection rates are closely monitored and also compared with comparable hospitals. […] In case our infection rates are lower, great, how can we further improve our performances? When our rates are higher, guys what is happening, what is going wrong here?”* (A5). Thus, a poor benchmark outcome motivates professionals to improve their safety performances and to learn from competitors.

Finally, hospitals’ attempts to differentiate themselves from competitors may also affect how they manage patient safety. In general, hospitals say they do not feel a strong need to differentiate themselves regarding patient safety, since patient safety is considered a basic requirement for providing healthcare. *“In our opinion, we should not compete for quality or safety, because the quality and safety should be guaranteed [in all hospitals], we do not want to use it for competitive advantage”* (D2). Nevertheless, hospitals did start to make a name for themselves. Two hospitals try, for example, to demonstrate greater openness and transparency than their competitors about the safety and outcomes of provided care. Moreover, most hospitals try to differentiate themselves by devoting attention to specific groups of patients. *“We pretend to be a hospital for elderly. Well, you cannot pretend this when your performance on the prevention of pressure ulcers is so disappointing.”* (C2). In line with this, all studied hospitals try to gain specific quality marks (e.g., for frail elderly) that may serve as a marketing tool for the care that the organisation delivers. So, the strategic choices of a hospital also influence their safety management.

### Experienced room to manoeuvre

How the dominant coalition deals with the institutional and competitive environment is influenced by the room to manoeuvre that a hospital experiences, which is in turn affected by hospital’s interpretation of safety requirements from external stakeholders as well as characteristics of the historically grown configuration of an organisation.

An important factor that influences the experienced room to manoeuvre is the tightness of external supervision. If external stakeholders impose more frequent or unexpected supervisory controls, hospitals face a higher risk of disclosure of non-compliance, leading to actions that might harm the organisation. Given the fact that hospitals want to reach accreditation, they experience, for example, little room to manoeuvre at the time of an accreditation visit; at that moment, they all try to perfectly meet the safety requirements. However, once a hospital is accredited, the experienced room to manoeuvre increases since the accreditation committee will not perform safety checks again until a next accreditation visit. As a nurse manager (A5) explained: “*In case of an accreditation visit, all of a sudden [all policies and procedures] are in order, but when the accreditation committee has left, everything collapses into a heap again”*. Comparably, departments in two of the studied hospitals were recently kept under close supervision of the Dutch Healthcare Inspectorate and experienced little room to manoeuvre: *“Our hospital has been checked by the Inspectorate and, at first, they did not give approval. […] Well, know that a manager visited our department and said make sure that everyone complies with all requirements, otherwise the hospital will be in big trouble”* (B5). In contrast, a recent positive evaluation could increase the experienced room to manoeuvre: *“Now that the Inspectorate is satisfied [with our performances] they may focus their attention to other hospitals”* (E3).

In addition, the experienced room to manoeuvre is also determined by the consequences of not meeting safety requirements (e.g., in terms of legitimacy or financial health). All studied hospitals feel a strong need to comply with requests made by health insurers, since the financial situation of a hospital is largely dependent on insurers’ willingness to purchase healthcare. *“For a while, I thought I am not going to respond [to all requests made by health insurers], but I have been rebuked by some members of the organisation who said, and they are right though, we have to get our money from that club*” (A2). In contrast, hospitals do also face external safety demands for which it is less obvious that the requirements have to be met. The consequences of not gaining a specific quality mark are, for example, less harmful for an organisation; thus, members of the dominant coalition experience more leeway to strategically choose whether they want to meet the criteria that such quality marks entail or not. *“Some quality marks are really important, but there are also a few that have little added value. […] Therefore, when a new quality mark is introduced we have to assess whether we want to gain it, […] what are the costs and what are the benefits?”* (A4).

The room to manoeuvre that the dominant coalition experiences is also influenced by the perceived relevance and practicality of demands that are imposed on the organisation. All studied hospitals employ a highly professionalised workforce that is socialised to strive for error-free care delivery and is professionally driven to improve patient safety. Hence, the more relevant a requirement is perceived to be, the less room to manoeuvre the dominant coalition experiences. *“If you are able to show that a lot of errors are made on a specific issue and that you have found a manner to actually avoid major errors, to avoid clinically relevant errors, then I think you will not hear anyone”* (D3). Thus, the perceived relevance depends on how serious safety problems are and how effective the safety requirements are perceived to be. Moreover, if hospitals face concrete and detailed safety requirements that can be easily incorporated in standard work processes they experience less room to manoeuvre.

Finally, the experienced room to manoeuvre is also affected by the historically grown configuration (i.e., the outcome of choices and responses to issues that the organisation had to deal with in the past). More specifically, it is influenced by the existence of a safety culture in which hospitals favour patient safety over other organisational aspects (e.g., production or finance). Some of the studied hospitals devote high priority to patient safety, because safety is closely linked with their organisational heritage or because of critical incidents in the past. A couple of years ago, one of the studied hospitals was, for example, confronted with media attention on hygiene problems as well as a persistent hospital infection. These incidents triggered awareness of patient safety and gave safety efforts new urgency and greater priority within the organisation. *“Of course, it was terrible that we were visited by a television show that used a hidden camera [which revealed hygiene problems], but it caused an enormous cultural change. […] Everyone was well aware that certain things had to change”* (E5). Hence, a culture was fostered in which the hospital strived for ongoing improvements in patient safety and nowadays the dominant coalition experiences more leeway to put their own spin on how they manage safety issues. This is in contrast with hospitals that are confronted with issues that distract their attention from patient safety, such as financial problems, a fall in production or a merger. Because of these issues, two of the studied hospitals gave priority to dealing with the financial situation of the organisation – “*Ninety percent of our time we talk about money and about budget cuts”* (B2). They experienced little room to manoeuvre; unless it would help them to save time that was spent on patient safety.

### Strategic responses

Depending on the room to manoeuvre that hospitals experience, the dominant coalition has a choice from various strategic responses (e.g., compliance, balancing or initiating change) on how they deal with external safety requirements. Whether the experienced room to manoeuvre is actually *utilized* depends on two things. First, the motivation and individual agency shown by members of the dominant coalition – in other words, do individuals have a personal drive to work on patient safety, do they feel responsible and do they dare to take a risk by deviating from external safety requirements. Second, the occurrence of safety incidents or near misses (i.e., unintended safety events that did not cause injury or damage to a patient, but that had the potential to do so) that trigger awareness for safety issues in the organisation at short notice.

The results of this study show that all studied hospitals comply with the majority of external demands regarding patient safety, both in terms of adopting safety practices or procedures and by providing required information for external accountability. However, different levels of compliance can be distinguished. In general, we found that hospitals fully comply with safety requirements if the directives are considered relevant and valuable for improving patient safety. *“Things like the surgical time-out procedure were imposed top-down, but they do contribute to reducing safety problems. They clearly cover a weak spot. […, so, that is something of which] we say, we just have to do it”* (D3). Full compliance with safety directives is also fostered by tight external supervision and serious consequences if requirements are not met. Moreover, it is facilitated if internal representatives of the various stakeholders actively support and stimulate the adoption of safety practices. Medical specialists who are in favour of certain safety improvements have, for example, an important role in gaining acceptance among their peers.

All studied hospitals also try to balance the useful directives of external stakeholders with the needs and practical experiences of their own employees, as they give healthcare professionals the opportunity to customise practices and procedures in order to fit the local circumstances. *“If really good arguments are presented of which healthcare professionals say this in particular makes things difficult, or we think we can arrange things better that way, […] then a protocol […] or procedure can be modified”* (C5). Modifications are mostly made in case of low practicality. Respondents argue, for example, that some of the evidence-based clinical protocols and guidelines issued by medical professional associations are so detailed and prescriptive that they do not always work out in practice. *“Clinical guidelines are rather frequently established by some kind of desk officers. These persons do work in hospitals, but often in academic centres which typically might be somewhat more precise in working conform evidence […]. However, maybe not always having medical practice in mind, especially of hospitals that treat a great amount of patients”* (E2). As a result, proposed safety requirements are not always in line with local circumstances in a hospital and may, consequently, lead to resistance to conform. Therefore, all studied hospitals offer their professionals the possibility to modify certain parts of the protocols and guidelines if they present good arguments to do so.

In addition, ceremonial implementation of safety requirements is used on a regular basis in all studied hospitals. Hospitals simply try to meet external requirements without fully acknowledging and internalising the need for these practices, because they are not so much willing or able to devote time and efforts to adopting certain practices. *“We noticed that, if we once again receive a new evaluation framework, we somewhat forced start ticking the boxes. […] A bit like we have to comply with this one, and this, and that, rather than thinking through the risks involved”* (E2). Ceremonial implementation is also demonstrated by required policies and procedures that do exist on paper, while the underlying changes in safety management or professional behaviours are not fully put into practice. *“On the outside, all policies and procedures show that we have things in order […], the bureaucrats here in the hallway do as much as they can. However, how are things experienced at the shop floor? Well, that is a problem”* (B2). This form of ceremonial implementation is chosen if supervisory agencies check whether hospitals established certain (written) procedures, of which healthcare professionals within the organisation consider the practical relevance to be low. Given the fact that organisations do not want to face sanctions, they choose for ceremonial implementation.

Overall, the studied hospitals do not give the impression that they often ignore or actively challenge safety demands. Even though hospitals do complain about the multitude and detail of safety requirements, they feel that it is almost impossible to abandon required practices and procedures because of the consequences of not meeting demands and since it is hard to offer collective resistance. However, on a small scale, some hospitals or departments do ignore safety requirements which they consider to be irrelevant. *“We had to develop a checklist on how to insert a central venous catheter line [in order to avoid infections …] but we had zero sepsis, for many years already! Then I said I am not going to make a checklist, I refuse to do so”* (D5). Moreover, some hospitals develop and discuss alternative approaches to mitigate identified safety risks: *“[Some safety procedures include] elements where we deliberately deviate from external requirements. […] We also discuss these things with the Dutch Healthcare Inspectorate, […] we just want to provide them with feedback on our practical experiences and how we arrange things differently”* (E2). Whether the dominant coalition undertakes such initiatives depends on the experienced room to manoeuvre. Hospitals that are highly dependent on approval of external stakeholders will not so easily challenge or ignore their requirements. In contrast, hospitals that recently received credits for their safety efforts and that give high priority to patient safety will more easily dare to stand out and will make more use of the experienced room to manoeuvre to challenge external safety requirements.

Finally, hospitals choose to take initiative in formulating and reshaping their safety management approach. Taking initiative requires room to manoeuvre and a pro-active role of members of the dominant coalition; characteristics that are often not so much fostered by external safety requirements. *“Organisations are increasingly pushed to take their own responsibility. However, this presupposes trust, whereas basically all imposed safety systems are created based on distrust”* (D2). Thus, initiating safety-related change assumes an intrinsic motivation to work on patient safety. In all studied hospitals, safety incidents or poor benchmark outcomes stimulate both healthcare professionals and members of the dominant coalition to implement safety policies and procedures that are not covered by or go beyond external requirements. *“We found out that, [compared to other hospitals], we had a higher chance of some kind of infection, which is really bad for a patient. Well, that launches a big drive to say we just have to set out very strict rules […], and we actually have to be even more strict than all those external requirements”* (E2). The degree to which further safety initiatives are developed varies across hospitals, based on the priority attached to patient safety and the level of individual agency shown by members of the dominant coalition. If hospitals have a culture which favours patient safety and when individuals in the organisation have a strong personal motivation, they take more initiative to put their own spin on how they manage several safety issues.

### Safety management approach

Different combinations of environmental conditions and strategic responses stimulate the adoption of either a control- or a commitment-based management approach. The dominant coalition tends to adopt a control-based management approach when they experience little room to manoeuvre and expect healthcare professionals to lack the intrinsic motivation to comply with safety requirements. Concrete and practicable safety requirements that are accompanied by tight external supervision and serious consequences when requisites are not met, are frequently incorporated in internal planning and control cycles and mostly give rise to a control-based management approach. *“Once every three months, we discuss the indicators [for which we are accountable to external stakeholders] with the board of directors. […] And if these indicators are not above the norm, then critical questions will be asked about it”* (C5). Especially, if professionals do not show full commitment to safety requirements and if compliance is not taken for granted, members of the dominant coalition monitor and control healthcare professionals’ behaviour. *“It all started with confidence that healthcare professionals would comply. Then we started monitoring, then we applied sanctions. There is pressure on it. It is mandatory. We impose controls and provide people with feedback”* (B5). In line with this, a control-based management approach is mostly used if the dominant coalition makes the strategic choice to comply with or ceremonially implement safety requirements. Finally, only in exceptional cases, where the dominant coalition experiences high urgency or strong pressure that healthcare professionals have to comply, sanction policies are used as part of a control-based approach. A business unit manager describes, for example, that they established sanction policies for hand hygiene compliance, because evidence had recurrently shown that good hand hygiene provides a sound basis for infection prevention. *“[When it comes to hand hygiene], you may push the boundaries twice, the third time you face a warning and the fourth time you will be fired. That is how important safety is for me. That is how much conforming to the norm is worth for me”* (A4).

In contrast, a commitment-based management approach is generally chosen if the dominant coalition expects safety requirements to generate an intrinsic motivation in healthcare professionals or when they experience plenty room to manoeuvre. If safety requirements are underlined by strong evidence or really target a clinically relevant issue, the dominant coalition typically assumes that a commitment-based management approach will effectively stimulate employees’ intrinsic motivation. Hence, the focus is on raising awareness of safety risks and explaining the relevance of safety practices. *“In the end, you want your patients to leave the hospital alive and healthy, they shouldn’t be harmed at all. So, I think that is the main motivation, often you only have to explain why you do certain things […] You have to talk a lot about safety matters”* (C3). Furthermore, the dominant coalition tends to adopt a commitment-based approach in case of safety demands that are difficult to put into concrete and controllable rules or regulations, and which therefore provide more room to manoeuvre. This is, for example, the case for so-called ‘soft skills’ such as speaking up behaviour. Speaking up behaviour is hard to enforce and the dominant coalition mostly tries to inspire healthcare professionals to express safety concerns or questions: *“On the one hand, you have to build awareness among nurses that they do have knowledge which they should use [in their collaboration with co-workers, in order to reduce safety risks], while on the other hand you should support them, show role modelling behaviour and emphasise that speaking up behaviour is something that we believe is really important”* (E5). Moreover, commitment-based management is used if the medical knowledge and specific expertise of healthcare professionals is needed to minimise safety risks or to put abstract external safety requirements into practicable safety procedures. *“As a manager, I can, of course, state that we score above or below a national average, but I cannot translate things into practical actions. What do we have to change in order to improve our safety performances? Well, that should really come from our employees, they have the expertise”* (B4). In these circumstances, the dominant coalition tries to stimulate healthcare professionals to proactively come up with new ideas for safety improvement by encouraging employees’ sense of ownership of patient safety and by actively inviting them to make safety recommendations. Finally, the adoption of commitment-based management approach does also require congruence with an organisational culture in which patient safety is prioritised at all organisational levels.

Even though control- and commitment-based management represent the opposite ends of a managerial spectrum, it never is an ‘either-or’ choice. Following the wide variety of institutional, competitive and configurational conditions as well as internal issues of strategic choice that organisations face, most hospitals simultaneously adopt elements of both management approaches or they alternately introduce elements of control- and commitment-based management in order to ensure patient safety. If the dominant coalition chooses, for example, to comply with safety requirements that they consider relevant, it depends on the pressure exposed by external stakeholders and the consequences that organisations face in case of non-compliance whether the balance shifts towards either a control- or a commitment-based management approach. The greater the pressure that hospitals face, the higher the chance that the dominant coalition chooses to monitor and control healthcare professional behaviours rather than relying on employees’ intrinsic motivation. Similarly, if healthcare professionals are offered the possibility to modify certain parts of externally exposed protocols or guidelines in order to make them fit local circumstances, the dominant coalition initially tries to inspire employees to work on patient safety and to encourage their sense of ownership. However, if experience shows that the modified safety requirements are not fulfilled in practice, the dominant coalition may also choose to combine a commitment-based management approach with elements of control, or to shift the balance entirely towards control-based safety management.

## Discussion

This study aimed to develop a deeper understanding of the effects of institutional, competitive and configurational factors as well as internal issues of strategic choice on the safety management approach of healthcare organisations. Results showed that, in all studied hospitals, general managers (e.g., board of directors, business unit managers and nurse managers) and medical specialists have a shared responsibility in decision-making processes on safety policies and practices. The choices that this dominant coalition makes while shaping safety management are strongly influenced by demands from stakeholders in the wider institutional environment and increasingly affected by competitive mechanisms deriving from the healthcare market. How the dominant coalition deals with these safety requirements is influenced by the room to manoeuvre that a hospital experiences. Little room to manoeuvre is experienced when hospitals face tight external supervision and serious consequences when safety requisites are not met or if concrete and detailed safety requirements are set that are perceived to be highly relevant. Under these circumstances hospitals will mostly choose a strategy of (passive) compliance; they just do what is required to be done. However, if safety demands are seen as irrelevant, hospitals sometimes choose a form of ceremonial implementation in which required policies and procedures do exist on paper, while the underlying changes in safety management or professional behaviours are not fully put into practice. More leeway is experienced if safety demands are abstract and the hospital has an organisational culture which favours patient safety. Hospitals will in these circumstances often try to balance internal and external demands, as they give healthcare professionals the opportunity to customise practices and procedures in order to fit the local circumstances. Hospitals do rarely ignore or challenge safety requirements, only when they perceive ample room to manoeuvre and safety requirements are either seen as irrelevant or very unpractical. The strategic choices hospitals make seem not only dependent on the experienced room to manoeuvre, but also on the motivation and individual agency of the dominant coalition. Hospitals that take their own initiative in formulating and reshaping their safety management approach are often those that experience leeway and in which members of the dominant coalition play a proactive role in prioritising patient safety. The occurrence of safety incidents or near misses can be an important trigger for this strategic response.

These strategic responses do, in turn, stimulate the adoption of either a control- or a commitment-based management approach. The dominant coalition tends to prefer a control-based approach when they experience little room to manoeuvre and expect healthcare professionals to lack intrinsic motivation. Thus, if hospitals face concrete and practicable safety requirements that lack clinical relevance, but that are accompanied by tight supervision and serious consequences if requisites are not met, direct supervisors frequently monitor and control healthcare professional behaviours. In contrast, the adoption of a commitment-based management approach is generally chosen if the dominant coalition expects safety requirements to generate intrinsic motivation in healthcare professionals or when they experience plenty of room to manoeuvre. Hence, if hospitals experience clinically relevant safety requirements or abstract requisites that are difficult to put into concrete and controllable regulations or require the specific expertise of healthcare professionals to transform them into practicable safety procedures, supervisors mostly focus on raising awareness of safety risks, explaining the relevance of safety practices and stimulating participation of healthcare professionals. Notwithstanding this dichotomy, following the wide variety of environmental conditions as well as internal issues of strategic choice that organisations face, all studied hospitals simultaneously or alternately apply elements of both management approaches in order to ensure patient safety.

**Fig. 2:**
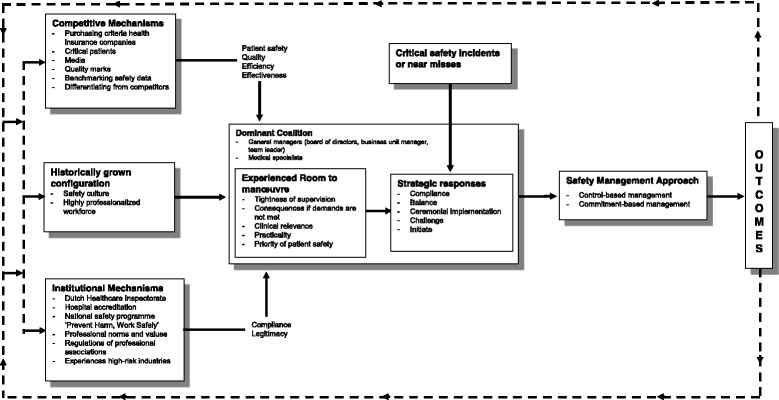
Framework for contextually based safety management in healthcare

By analogy to the contextually-based HR theory [11, 12], we established a framework for shaping safety management in healthcare (see Fig. 2). In this sector, medical specialists have a prominent role in shaping safety management, alongside managers and other staff. Despite the fact that managers’ sphere of influence has been extended over the last years, healthcare professionals still remain highly influential when it comes to their clinical work and when their specific expertise is essential for shaping effective practices and procedures [39]. Ensuring patient safety has, thus, become a shared responsibility of general managers and healthcare professionals. Secondly, our findings add to the original framework that, in case of patient safety, incidents or near-misses frequently lead to ad-hoc modifications in safety policies and procedures. In HR management, critical incidents and organisational scandals have been found to affect the administrative heritage and accordingly influence the shaping of HR practices and procedures [40]. Yet, in case of patient safety, incidents typically induce short-term learning processes in which organisations investigate what happened and make changes in care processes or safety management in order to reduce the probability of recurrence of similar events. As a consequence, safety incidents or near-misses are important triggers for (re)shaping safety management on short notice. Finally, several feedback loops between the environmental conditions and the strategic choices of the dominant coalition are to be expected. Poor safety outcomes may, for example, not only lead to ad-hoc modifications in safety management but also give rise to new rules and regulations established by medical professional associations [41, 42]. Furthermore, strategic responses of the dominant coalition may also provoke reactions of external stakeholders. If the dominant coalition chooses to challenge or ignore external safety requirements, stakeholders may tighten their supervision or broaden consequences when demands are not met.

The institutional and competitive conditions presented in this study show that, in order to ensure safe care delivery, external stakeholders often impose detailed safety requirements, strong forces for compliance and growing demands for accountability. These external regulations have focused hospitals’ attention on patient safety and they have led to intensified efforts to reduce safety incidents. However, strict safety requirements may also have disadvantages. A strong focus on externally regulated compliance and transparency generates extrinsic motivation in employees but it may, at the same time, undermine or even diminish intrinsic motivation to work on patient safety [43]. This is further reinforced by the control-based management approach that is generally preferred if hospitals face great pressures from external stakeholders. A control-based approach does strengthen employees’ extrinsic motivation by providing directions and punishing or rewarding employee behaviours [44]. It is however contradictory to management control systems that are traditionally used in professional organisations, which are typically based on the intrinsic motivation and professional autonomy of healthcare professionals [45]. Furthermore, emphasis on compliance seems to lead to situations in which some hospitals become primarily concerned with conformity to external safety requirements, rather than proactively dealing with safety risks that are important to the organisation [46]. As a consequence, external regulations may help to keep healthcare safe, but they may also impede progress beyond a certain level [4]; especially in organisations that do prioritise patient safety and that spontaneously strive for excellence. Fostering a proactive safety culture would require a more trust-based control system and ample room to manoeuvre [46]. The Dutch Healthcare Inspectorate and health insurers have recently launched initiatives along these lines. They started introducing systems of so-called ‘horizontal inspection’ in which organisations are granted exemption from tight supervision after they have proven that self-regulations ensures adequate (safety) performances [47, 48]. Thus, external stakeholders have made some first attempts to rely more on trust rather than tight controls, which may, in turn, reinforce the adoption of a commitment-based safety management approach, increase intrinsic motivation in healthcare professionals and stimulate hospitals to proactively deal with safety risks.

This study has some limitations that support the need for future research. First, only respondents in managerial positions or with a leading role in safety management within hospital organisations were interviewed. The focus on intra-organisational actors is consistent with the explorative nature of this study and our aim to gain insight into how organisations shape their safety management approach. However, in future research, it may be interesting to include external stakeholders that impose safety requirements on hospitals. This may help to gain broader insight into the institutional and competitive mechanisms that influence hospitals’ safety management approach by identifying conditions that are overlooked by intra-organisational actors (e.g., horizontal inspection) and it may help to develop understanding of reciprocity between organisational responses and conditions in the wider hospital environment (i.e., feedback loops in our model). Second, the study exclusively focused on hospitals in the Netherlands. Therefore, the generalizability to other healthcare-contexts or other countries may be low. However, Dutch hospitals can also be considered an interesting case because they are subject to safety demands from a diverse set of stakeholders in the institutional and competitive environment [2], and they managed to achieve a considerable reduction in preventable deaths over the previous few years [49]. Future research may examine which (combination of) management approach(es) contributes to the achievement of this result and, more in general, what the effects of control- and commitment-based management are on patient safety.

## Conclusions

In conclusion, patient safety management requires a balanced approach in which hospitals are encouraged to combine both control- and commitment-based management practices. Institutional and competitive pressures as well as strategic choices that hospitals make, result in various combinations of the safety management approaches. The dominant coalition tends to prefer a control-based approach when they experience little room to manoeuvre and when they expect healthcare professionals to lack intrinsic motivation. The adoption of a commitment-based management approach is generally chosen if the dominant coalition expects safety requirements to generate intrinsic motivation in healthcare professionals of when they experience plenty of room to manoeuvre. External pressures mainly steer managers towards a control-based safety management approach, which generates extrinsic motivation in employees but may, at the same time, undermine or even diminish intrinsic motivation to work on patient safety. Hence, external stakeholders should balance strong forces for compliance with more trust-based safety demands, consequently giving rise to both control- and commitment-based safety management approaches.

## Abbreviation

HR: Human resources
